# Expression of Human Frataxin Is Regulated by Transcription Factors SRF and TFAP2

**DOI:** 10.1371/journal.pone.0012286

**Published:** 2010-08-20

**Authors:** Kuanyu Li, Anamika Singh, Daniel R. Crooks, Xiaoman Dai, Zhuangzhuang Cong, Liang Pan, Dung Ha, Tracey A. Rouault

**Affiliations:** 1 Jiangsu Key Laboratory for Molecular Medicine, Medical School of Nanjing University, Nanjing, China; 2 Molecular Medicine Program, National Institute of Child Health and Human Development, Bethesda, Maryland, United States of America; 3 Department of Biochemistry, Molecular and Cellular Biology, Georgetown University Medical Center, Washington, D. C., United States of America; Virginia Commonwealth University, United States of America

## Abstract

**Background:**

Friedreich ataxia is an autosomal recessive neurodegenerative disease caused by reduced expression levels of the frataxin gene (*FXN*) due to expansion of triplet nucleotide GAA repeats in the first intron of *FXN*. Augmentation of frataxin expression levels in affected Friedreich ataxia patient tissues might substantially slow disease progression.

**Methodology/Principal Findings:**

We utilized bioinformatic tools in conjunction with chromatin immunoprecipitation and electrophoretic mobility shift assays to identify transcription factors that influence transcription of the *FXN* gene. We found that the transcription factors SRF and TFAP2 bind directly to *FXN* promoter sequences. SRF and TFAP2 binding sequences in the *FXN* promoter enhanced transcription from luciferase constructs, while mutagenesis of the predicted SRF or TFAP2 binding sites significantly decreased *FXN* promoter activity. Further analysis demonstrated that robust SRF- and TFAP2-mediated transcriptional activity was dependent on a regulatory element, located immediately downstream of the first *FXN* exon. Finally, over-expression of either SRF or TFAP2 significantly increased frataxin mRNA and protein levels in HEK293 cells, and frataxin mRNA levels were also elevated in SH-SY5Y cells and in Friedreich ataxia patient lymphoblasts transfected with SRF or TFAP2.

**Conclusions/Significance:**

We identified two transcription factors, SRF and TFAP2, as well as an intronic element encompassing EGR3-like sequence, that work together to regulate expression of the *FXN* gene. By providing new mechanistic insights into the molecular factors influencing frataxin expression, our results should aid in the discovery of new therapeutic targets for the treatment of Friedreich ataxia.

## Introduction

Friedreich's ataxia, the most common inherited ataxia, is an autosomal recessive neurodegenerative disease caused by expansion of triplet nucleotide GAA repeats in the first intron of the *FXN* gene. Expansion of the GAA region from fewer than 200 to as many as 1500 repeats results in significant reduction of frataxin protein levels in affected patient tissues. The exact physiological function of frataxin continues to be a subject of intense research. Early reports demonstrated robust mitochondrial iron accumulation in Friedreich ataxia patient cardiac tissue [1], as well as in a *Saccharomyces cerevisiae* strain lacking the yeast frataxin homologue Yfh1p [2]. Additionally, deficiency of iron-sulfur (Fe-S) cluster-containing mitochondrial respiratory chain enzymes is a feature found both in patient cardiac biopsies and in Yfh1p-deficient *S. cerevisiae* [3]. These seminal findings regarding frataxin function have led to further work suggesting potential roles for human frataxin (and its homologues in lower organisms) in cellular functions including as an iron donor for heme biosynthesis [4], as an iron storage protein [5], as an iron chaperone [6] or accessory protein [7] important for Fe-S cluster assembly. Although there is ongoing debate over the function(s) of frataxin, it seems clear that its absence in human cells results in impaired Fe-S protein activities as well as mitochondrial iron overload.

The clinical manifestations of Friedreich ataxia involve neurodegeneration in the spinal cord and cerebellum, causing gait disturbances, speech impairment, and increased incidence of diabetes. Mitochondrial iron deposition in the heart is known to accompany the hypertrophic cardiomyopathy and eventual heart failure observed in Friedreich ataxia patients, which commonly leads to mortality in the third or fourth decade of life (reviewed elsewhere [8]). Since oxidative tissue damage is thought to result from mitochondrial iron overload, drug screening studies have focused on ameliorating cardiac iron accumulation using iron chelators [9], [10], and enhancing respiratory chain function using coenzyme Q10 and/or reducing oxidative damage with antioxidants [11], [12]. The effectiveness of these treatments in improving cardiac and neurological outcomes in Friedreich ataxia patients is under continued evaluation.

A recent study demonstrated an association between the GAA repeats within the *FXN* gene and aberrant frataxin pre-mRNA processing [13], and the authors proposed that binding of transcribed GAA repeats to nuclear splicing factors can interfere with turnover of intronic RNA and lead to decreased abundance of mature mRNA [13]. However, accumulating evidence indicates that epigenetic changes caused by heterochromatin formation in the promoter region and/or the first intron of the *FXN* gene also contribute to the dramatic reduction of frataxin protein levels in Friedreich ataxia patients. Decreased histone acetylation and extensive methylation of CpG regions upstream of the GAA repeat are observed in Friedreich ataxia patient cell lines and tissues [14], [15], suggesting that enhanced heterochromatin formation might impede the transcription of frataxin, leading to lower frataxin protein levels [14], [16], [17]. Recently, a study employing an experimental histone deacetylase (HDAC) inhibitor in a mouse model of Friedreich ataxia revealed that this drug can substantially increase frataxin mRNA and protein levels [16]. Reduction of frataxin transcription very likely results from reduced accessibility of transcriptional regulatory factors to the promoter region and/or trinucleotide repeat region [15], [18], [19], [20]. However, the identity and number of the regulatory factors influencing frataxin expression are largely unknown. Thus, in-depth investigation of the transcriptional regulatory machinery involved in frataxin expression would aid in the identification of drugs or therapies directed at restoring frataxin protein levels in Friedreich ataxia patient tissues.

In this study, we used bioinformatic and molecular techniques to identify two transcription factors, SRF and TFAP2, which directly bind to the promoter region of the *FXN* gene. TFAP2 up-regulated frataxin mRNA expression in several cell lines, whereas SRF showed cell-line specific influences on frataxin expression. Finally, over-expression of either transcription factor in Friedreich ataxia patient-derived lymphoblasts or cell lines significantly increased frataxin mRNA levels. Identification and further characterization of these two new factors involved in frataxin expression may aid in the development of new therapeutic avenues for the treatment of Friedreich ataxia.

## Results

**Figure 1:**
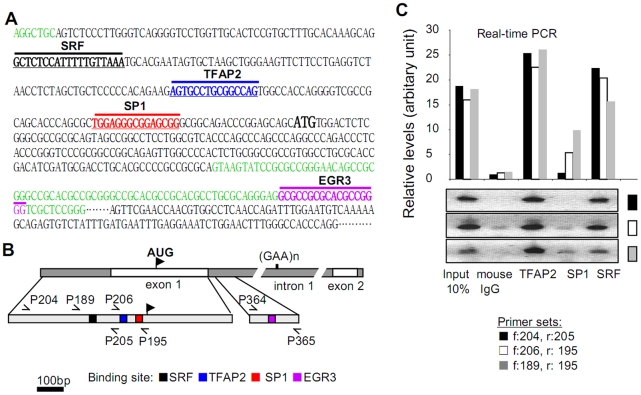
Transcription factors SRF and TFAP2 bind to the *FXN* gene promoter region *in vivo*. (A) Sequence of 5′-end of the human *FXN* gene. Sequences are color-coded: intronic sequences in green, exon 1 and exon 2 in black. Putative transcription factor binding sites identified by Genomatix analysis are annotated in bold black for SRF, in bold blue for TFAP2, in bold red for SP1, and in bold pink for EGR3. Start codon (AUG) in exon 1 is in bold black. (B) Schematic diagram of human *FXN* promoter region showing the location of the putative regulatory sequences. Black-filled square: SRF binding site; blue-filled square: TFAP2 binding site; red-filled square: SP1 binding site; pink-filled square: EGR3 binding site. Primers used in the ChIP assays are designated as: P189, P195, P204, P205, P206, P364, and P365. (C) Binding of SRF and TFAP2 to the *FXN* promoter *in vivo* was quantified by chromatin immunoprecipitation (ChIP) and quantitative real-time PCR (qRT-PCR). The upper panel is the quantification of *FXN* promoter chromatin, immunoprecipitated using antibodies raised against SRF, TFAP2, and SP1. Three pairs of primers flanking the putative binding sites of these transcription factors were used for the qPCR analysis. The lower panel shows the end-products of the qPCR reactions separated by agarose gel electrophoresis. Primers specific for human *FXN* intron 4 were included as an internal negative control (data not shown).

In previous work we observed significant decreases in frataxin mRNA levels in multiple human cell lines as well as primary human fibroblasts and lymphoblasts derived from Friedreich ataxia patients and controls, when treated with the iron chelator desferrioxamine (DFO) [21]. These data suggested that one or more regulatory elements might modulate transcription of frataxin under varying metabolic conditions such as iron starvation. We initiated the current study by using Genomatix software (www.genomatix.de) to identify putative transcription factor binding sites within the promoter that might serve as transcriptional regulatory elements of the *FXN* gene. Although the *FXN* promoter region was reported to extend at least 1255 bp upstream of the translation start site (AUG) [22], *in vitro* experiments suggested that more than 60% of *FXN* promoter activity is conferred by the first 221 bp of this upstream sequence [22]. We chose to pursue putative SRF (serum response factor), TFAP2 (transcription factor AP2), and SP1 binding sites within this 221 bp region due to a high matrix similarity score of greater than 0.95 for these sequences (Figure 1A). SRF binds to sequences known as serum response elements, and is a member of the MADS (MCM1, Agamous, Deficiens, and ARF) box superfamily of transcription factors that can stimulate cell proliferation and differentiation [23]. TFAP2 is a developmentally regulated, retinoic-acid inducible transcriptional activator [24], while SP1 is a ubiquitously expressed factor that interacts with numerous other transcription regulators involved in many pathways (e.g., HIV-1 Tat, P53, RNAPII, EGR1, TFAP2) [25], [26]. Notably, the SRF and TFAP2 binding sites were found within-, and immediately downstream of one of the few regions of the *FXN* promoter that shows significant sequence similarity between rodents and primates due to the presence of an L2 retrotransposon-like sequence [22].

To test whether SRF, TFAP2 or SP1 bind to the predicted *FXN* promoter sequence elements defined above, chromatin immunoprecipitation (ChIP) was performed using anti-SRF, TFAP2, and SP1 primary antibodies, and qRT-PCR was used to quantify the degree of transcription factor binding in the *FXN* promoter region. Robust SRF and TFAP2 binding was observed in HEK293 cells using several qRT-PCR primer sets (Figure 1B) encompassing the promoter region of *FXN*, while binding of SP1 was found to be far weaker than that of SRF or TFAP2 (Figure 1C). Similar results were obtained for each primer set (Figure 1C, lower panel) and comparable results were also obtained using K562 erythroleukemia cells (not shown).

**Figure 2:**
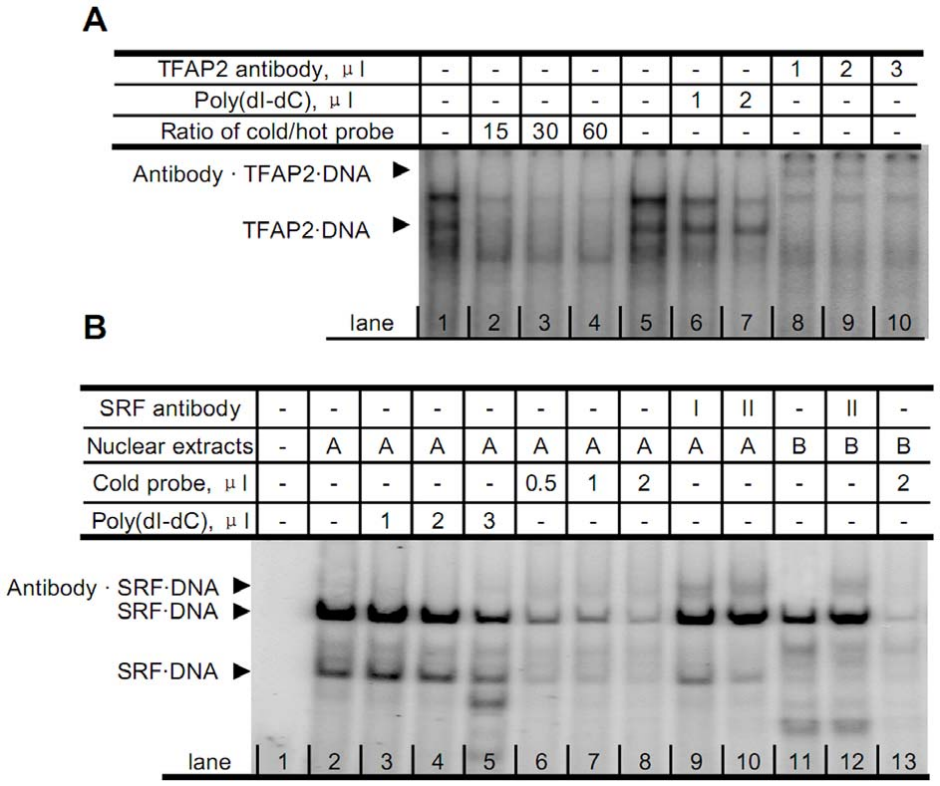
Transcription factors SRF and TFAP2 bind to the *FXN* promoter region *in vitro*. EMSA analysis was performed to investigate binding of TFAP2 (A) and SRF (B) to the promoter region of *FXN in vitro*. Nuclear extracts from HEK293 cells were incubated with [γ-^32^P]-ATP labeled oligonucleotides coding the predicted TFAP2 or SRF binding site on the *FXN* promoter region of interest for 1 hour at 4°C. The binding products were resolved in native polyacrylamide gels (see MATERIALS AND METHODS). Specific competitor (non-radioactive oligonucleotide, so called cold probe, 10 µM) or non-specific competitor (poly(dI-dC), 0.5 mU/µL) was added to assess the specificity of the binding of SRF or TFAP2. Antibodies of TFAP2 (2 mg/ml) and SRF (0.5 mg/ml) were added for supershift, respectively. I: SRF antibody purchased from Santa Cruz Biotech.; II: SRF antibody purchased from Active Motif. A: HEK293 cell nuclear extracts, prepared by the authors, B: Jurkat cell nuclear extracts, purchased from Active Motif (Carlsbad, CA).

Next, *in vitro* electrophoretic mobility shift assays (EMSA) were carried out to assess the binding of nuclear proteins to synthetic dsDNA oligonucleotides encoding the putative TFAP2 and SRF sequences found in the *FXN* promoter. *In vitro* binding of nuclear proteins to the TFAP2 and SRF sequences was observed (Figure 2A, 2B). Moreover, when antibodies raised against either TFAP2 or SRF were included in the reactions, a ‘supershift’ was observed (Figure 2A lane 8, 9, 10 and 2B lane 9, 10, 12), suggesting formation of specific complexes between SRF or TFAP2 and the respective antibodies, resulting in altered mobility within the gel. These results strongly suggest that both SRF and TFAP2 can bind to specific sequences within the promoter region of the *FXN* gene.

**Figure 3:**
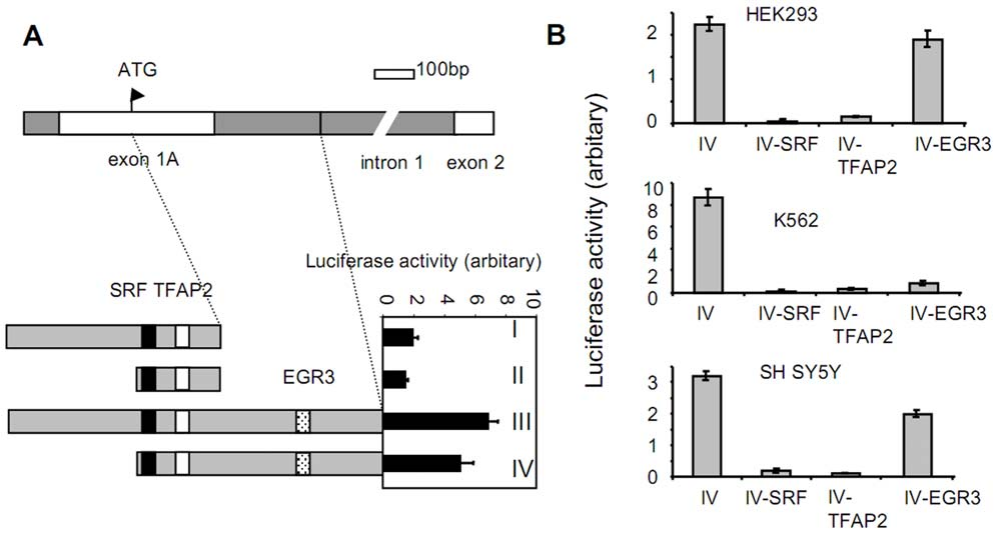
The SRF and TFAP2 binding sites in the *FXN* promoter are important for frataxin expression. (A) Luciferase analysis of a novel intronic regulatory region of the *FXN* gene. The upper panel portrays the upstream region of the *FXN* gene including the exon 1 and 2 and intron 1. Bottom panel: luciferase activity was measured in cells transfected with luciferase constructs containing truncated *FXN* promoter fragments containing the SRF and TFAP2 binding sites. Filled square: SRF binding site; open square: TFAP2 binding site; dotted square: EGR3 binding site. The four luciferase constructs are designated as I, II, III, IV (see MATERIALS AND METHODS). (B) Mutation of the SRF and TFAP2 binding sites in the *FXN* promoter dramatically decreased luciferase activity driven from *FXN* promoter fragment IV. Mutation of the predicted EGR3 transcription factor binding site in intronic sequence of the *FXN* gene showed cell line-specific effects on transcriptional activity. Three separate experiments were carried out. For each experiment, duplicate transfections were performed. Error bars represent the standard deviation.

Transcriptional regulatory elements have previously been identified within the first intron of several genes [27], [28], [29], [30]. Recently a region immediately upstream of the intronic GAA expansion which was reported to be important for maximal frataxin expression [15]. Further experiments showed that expansion of the GAA repeats leads to changes in DNA methylation and chromatin structure in this region of the *FXN* gene [15]. Accordingly, we analyzed the region immediately downstream of exon 1 using Genomatix software, and found a putative EGR3 transcription factor binding site in this intronic region (Figure 3A). To investigate whether this putative EGR3 binding site and the identified SRF and TFAP2-binding sites are involved in transcription of frataxin, we generated four luciferase reporter constructs. The first two constructs contained promoter fragments that extended 558-bp (construct I) or 228-bp (construct II) 5′ of the start codon AUG linked to the luciferase gene; the latter two (constructs III and IV) contained the same promoter regions as construct I and II, respectively, but they also contained part of the first intron including the proposed EGR3 binding site (GCGCCGCGCACGCCGGG) (Figure 3A, dotted square). The insert sizes were 1030-bp (III) and 650-bp (IV), separately. Luciferase activity from constructs III and IV was substantially higher than activity measured from constructs I and II (Figure 3A, bottom panel), suggesting that the intronic region downstream of exon 1 is important for transcription of the *FXN* gene.

We next used the luciferase assay to probe promoter activity of variants of the above constructs in which the putative binding sites for SRF, TFAP2, or EGR3 were altered. Mutation of either the SRF or TFAP2 binding sites in construct IV resulted in dramatically decreased luciferase activity in three distinct cell lines (Figure 3B). In contrast, minimal decreases in luciferase activity were observed following mutagenesis of the SRF or TFAP2 binding sites in constructs I and II (not shown), which lack the intronic sequence downstream of exon 1 that is included in construct IV. Interestingly, mutagenesis of the EGR3 binding site in the *FXN* intronic sequence in construct IV had dramatically different effects on luciferase expression depending on the cell lines tested, causing severe, mild, or no significant effect on the promoter activities in K562, SH SY5Y, and HEK293 cells, respectively. Taken together, these results further suggest that SRF and TFAP2 are transcription factors important for frataxin expression, and the intronic sequence downstream of exon 1 which contains a putative EGR3 binding site is required for full expression of frataxin.

**Figure 4:**
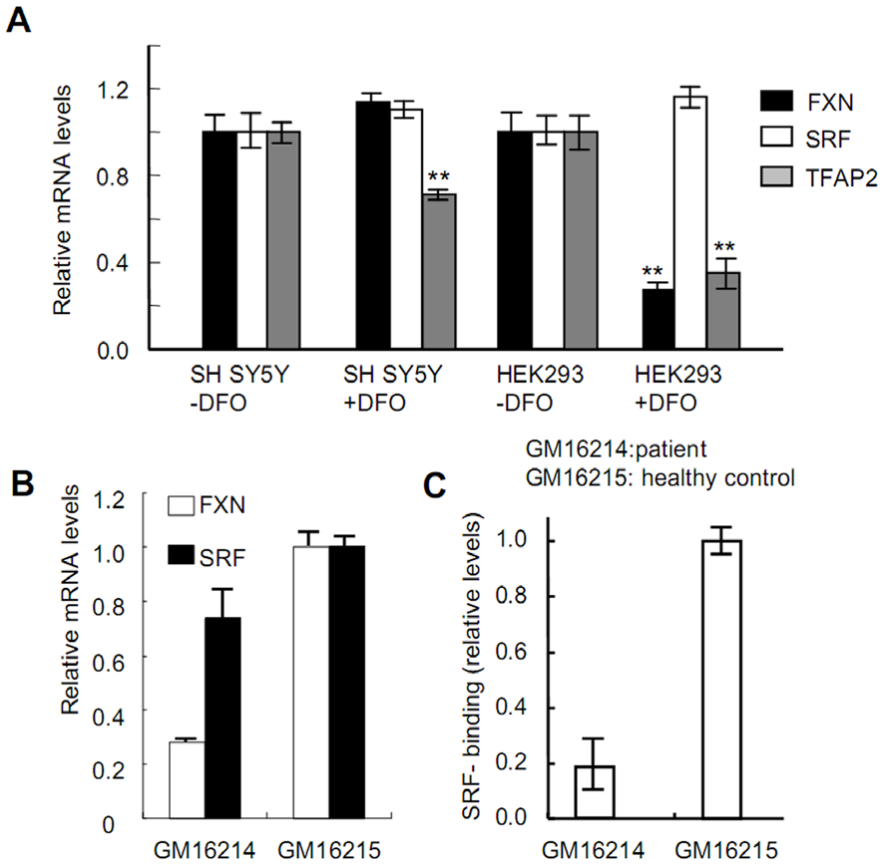
Cellular iron depletion decreases frataxin expression. (A) Iron depletion decreases frataxin and TFAP2 mRNA levels in tested cells. qRT-PCR was performed to quantify the mRNA levels of frataxin, SRF, and TFAP2 in SHSY5Y and HEK293 cells following treatment with 30 µM DFO for 48 hours. GAPDH was used as an internal control. Three separate experiments were carried out. For each experiment, duplicate iron treatment was performed. The error bars represent standard deviation. Statistical analysis was performed using the Student's *t*-test: **: ρ<0.001. (B) mRNA levels of SRF and frataxin in lymphoblasts derived from patient (GM16214) and healthy control (GM16215). qRT-PCR was performed to quantify the mRNA levels of frataxin and SRF with the total RNA isolated from patient and control. (C) Binding of SRF to the *FXN* promoter is dramatically decreased in patient lymphoblasts. ChIP assays were conducted as described in Figure 1. The relative expression level in the patient cells is normalized to the healthy control cells, which are set at 1.

Previously, we demonstrated that frataxin protein and mRNA transcript levels are altered by perturbations in cellular iron status brought on by treatment with the iron chelator DFO [21]. To test whether iron-mediated changes in frataxin transcript levels might be related to altered expression of either SRF or TFAP2, we treated HEK293 and SH SY5Y cells with DFO and measured mRNA levels of SRF and TFAP2 by qRT-PCR. While no significant change in SRF mRNA levels was observed in iron-depleted (+DFO) versus iron-replete (-DFO) cells, TFAP2 mRNA levels were significantly decreased by iron depletion in both HEK293 and SH SY5Y cells (Figure 4A). Frataxin mRNA levels were also decreased by DFO treatment in HEK293 cells (as reported previously [21]), but not in SH SY5Y cells (Figure 4A). These data suggest that iron-mediated alterations in TFAP2 expression levels might influence frataxin mRNA expression during cellular iron deficiency.

We also checked the mRNA expression levels of SRF, TFAP2, and EGR3 in lymphoblasts derived from an Friedreich ataxia patient (GM16214) and from a healthy control (GM16215). We found that EGR3 expression was very low, while TFAP2 expression was undetectable (not shown). As expected, frataxin mRNA levels were reduced in the Friedreich ataxia patient lymphoblasts as compared to the control lymphoblasts, while SRF mRNA expression levels were only moderately different between the two cell lines (Figure 4B). Finally, a ChIP experiment revealed that SRF occupancy of the *FXN* promoter was reduced in the Friedreich ataxia patient lymphoblasts as compared to the control lymphoblasts (Figure 4C).

**Figure 5:**
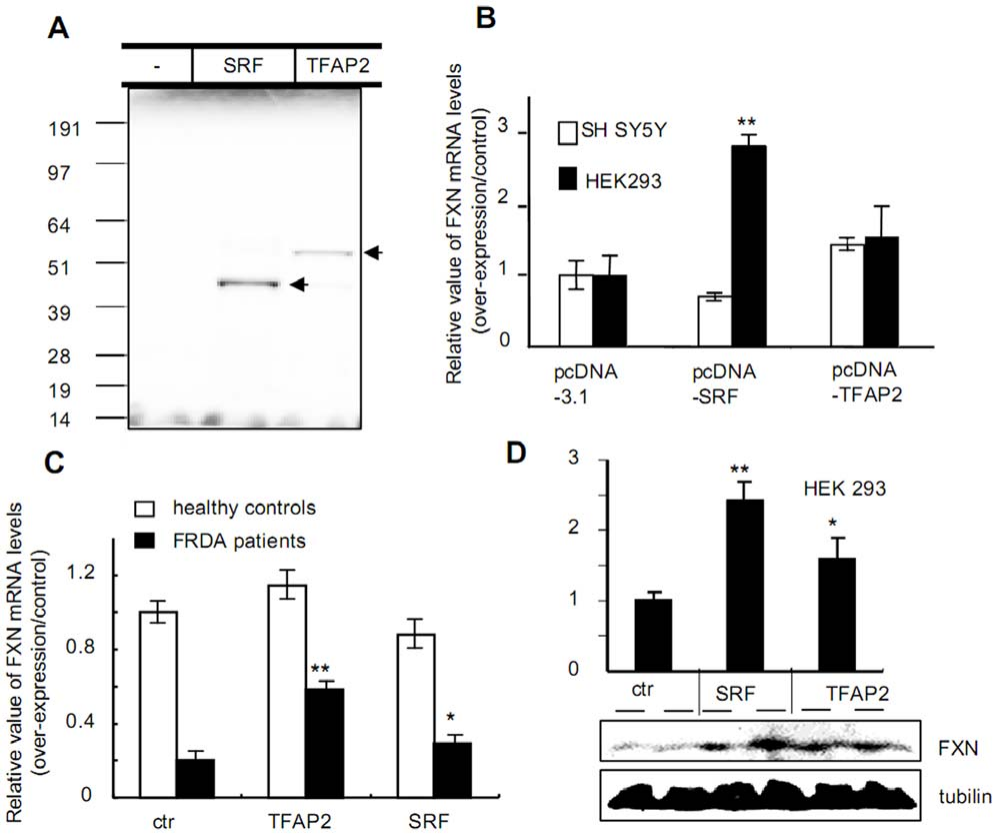
Overexpression of SRF or TFAP2 enhances frataxin expression in HEK293 cells and Friedreich ataxia patient lymphoblasts. (A) In order to assess the effect of over-expression of SRF or TFAP2 on frataxin expression, plasmids pcDNA-SRF and pcDNA-AP2 were constructed and the correct sizes of the translation products of the cloned SRF and TFAP2 cDNA were verified by *in vitro* translation with [^35^S]-labeled methionine. The plasmid constructs were then transfected into HEK293 or SH SY5Y cells (B), or lymphoblasts (C) derived from healthy individuals (GM15799, GM16215) or Friedreich ataxia patients (GM16179, GM16214), and frataxin mRNA levels were measured by qRT-PCR (see MATERIALS and METHODS). Empty plasmid pcDNA3.1(-) was used as a control for comparison. The results from two different experiments were similar; the data presented here are average values from the two experiments. Levels of mature frataxin protein were determined by western blot (D). A representative western blot is shown here for HEK293 cells. Three separate experiments were carried out, and for each experiment two transfections per sample were performed. Statistical analysis was performed using the Student's *t*-test: *: ρ<0.05; **: ρ<0.001.

The above data suggest that frataxin expression could be enhanced by heterologous expression of SRF or TFAP2. To test this hypothesis we over-expressed SRF or TFAP2 in SH SY5Y cells, HEK293 cells, and in lymphoblasts obtained from an Friedreich ataxia patient or from a healthy control individual, and qRT-PCR was performed to assess changes in cellular frataxin mRNA levels following transfection (Figure 5). First, we performed *in vitro* translation to verify that our plasmid constructs expressed SRF and TFAP2 protein products of the correct size (Figure 5A). SRF and TFAP2 mRNA levels were found to be more than 100-fold higher in cells transfected with plasmids pcDNA-SRF or pcDNA-TFAP2 than in control cells transfected with an empty plasmid control, pcDNA3.1(-) (data not shown). Over-expression of SRF in HEK293, but not SH-SY5Y cells resulted in significantly elevated frataxin mRNA levels, while over-expression of TFAP2 in either cell line resulted in modest increases in frataxin mRNA levels (Figure 5B). Strikingly, over-expression of either TFAP2 or SRF in Friedreich ataxia patient lymphoblasts resulted in significant increases in frataxin mRNA levels, while over-expression of either transcription factor in control lymphoblasts had no significant effect on frataxin mRNA levels (Figure 5C). Finally, we measured frataxin protein levels in HEK293 cells after transfection with either SRF or TFAP2. Consistent with the changes in frataxin mRNA levels observed in these cells (Figure 5B), western blots demonstrated that frataxin protein levels were also increased (Figure 5D). Together, these results suggest that frataxin expression levels can be influenced by both SRF and TFAP2 expression in a variety of cell types.

## Discussion

Deficiency of the frataxin protein is the primary molecular defect in Friedreich ataxia disease. Frataxin levels in Friedreich ataxia patients vary from between 5% and 30% of normal levels, while healthy heterozygous carriers typically express more than 50% of normal frataxin levels [31], [32], [33]. Thus, it has been suggested that restoration of frataxin gene expression levels in Friedreich ataxia patients to levels observed in heterozygotes may substantially slow disease progression. Characterization of the regulatory elements controlling frataxin expression is critical in the development of therapies directed at restoring frataxin expression levels in Friedreich ataxia patients. In this study we identified two transcription factors, SRF and TFAP2, which directly bind the promoter region of the human *FXN* gene and alter frataxin mRNA and protein levels. Over-expression of either of these two transcription factors led to a significant increase in frataxin mRNA levels in Friedreich ataxia patient lymphoblasts. Furthermore, frataxin protein levels were increased following over-expression of SRF or TFAP2 in HEK293 cells. Thus, we conclude that transcription factors SRF and TFAP2 directly influence frataxin expression.

We previously demonstrated that cells derived from Friedreich ataxia patients show signs of cytosolic iron deficiency, and that cellular frataxin mRNA levels were decreased after experimental induction of iron deficiency [21]. Here we have demonstrated that mRNA levels of the transcription factor TFAP2, but not SRF, are also regulated by iron in HEK293 cells. In affected tissues of Friedreich ataxia patients, cytosolic iron depletion brought on by decreased frataxin expression due to expansion of the GAA repeat might result in decreased expression of TFAP2, resulting in an even further reduction in frataxin expression. Unexpectedly, overexpression of TFAP2 did not significantly increase mRNA levels of frataxin in SH-SY5Y, HEK293, or lymphoblasts derived from healthy control individuals. However, frataxin expression levels were significantly enhanced by TFAP2 over-expression in the Friedreich ataxia patient lymphoblast cell line. These effects may be a result of different levels of occupancy of endogenous TFAP2 on the putative TFAP2 binding site in the frataxin promoter. Additionally, the TFAP2 family of transcription factors is composed of five distinct gene products in humans (AP-2α, AP-2β, AP-2χ, AP-2δ, and AP-2ε), which can heterodimerize and elicit tissue-specific transcriptional regulatory effects [24]. Therefore, heterologous expression of additional TFAP2 family members may be needed in order to see positive effects on frataxin expression in all cell types utilized in this study.

Many genes contain intronic regulatory sequences that are important for gene expression [34], [35]. Bisulfite sequence mapping of the region immediately upstream of GAA repeats within *FXN* intron 1 (724 bp) identified sequences that enhance *FXN* promoter activity [15]. However, this prior study did not address the function of upstream sequences found immediately following exon 1 (530 bp away from the 724 bp fragment). We observed diminished luciferase activity in *FXN* promoter constructs that lack this intronic region (Figure 3A; constructs I and II), which contains a predicted consensus binding site for the EGR3 transcriptional regulator. Since distinct band shift patterns were observed in further EMSA experiments performed with nuclear extracts from K562 and SH SY5Y cells (data not shown), we may speculate that SRF and TFAP2 interact with other regulatory elements to modulate frataxin expression in a cell-type dependent manner. EGR3 is a good candidate co-regulator, since mutagenesis of the predicted EGR3 binding site in our luciferase experiments resulted in variable enhancer effects in the different cell lines that were tested (Figure 3B). Interestingly, EGR3-deficient mice exhibit several neuromuscular defects including gait ataxia and scoliosis [36], symptoms which resemble those exhibited by Friedreich ataxia patients. Moreover, EGR1 and EGR3 double knockout mice displayed decreased frataxin mRNA levels (57.3% of control) in thymocytes [37], indicating that loss of EGR1/3 can transcriptionally alter frataxin expression *in vivo*.

In conclusion, we have identified two nuclear transcription factors, SRF and TFAP2, which can directly bind sequences in the promoter of the human *FXN* gene, likely enhancing frataxin expression. Expression of recombinant transcription factors SRF or TFAP2 in two distinct human cell lines, as well as in Friedreich ataxia patient lymphoblasts, resulted in increased frataxin mRNA levels. These results demonstrate that frataxin expression can be enhanced by these two key transcription factors. Potential interaction partners of SRF and TFAP2, including EGR3, should be further explored to shed light on the regulatory network governing frataxin expression, as well as the tissue-specific pathology of Friedreich ataxia disease.

## Materials and Methods

### Cell culture and transfection

Cell lines HEK293, K562, and SH SY5Y were purchased from ATCC (Manassas, VA). Lymphoblasts derived from healthy controls (GM15799, GM16215) and Friedreich ataxia patients (GM16197, GM16214) were obtained from the Coriell Cell Repository (Camden, NJ). HEK293 cells were grown in alpha-modified MEM medium (Sigma, St. Louis, MO), SH SY5Y in DMEM/F-12 medium (Invitrogen, Carlsbad, CA), and lymphoblasts in RPMI 1640 medium (Invitrogen), all supplemented with 10% fetal calf serum and 2 mM glutamine. For transfection of HEK293 and SH SY5Y cells, Fugene 6 (Roche, Indianapolis, IN) was used according to the supplier's manuals. For transfection of lymphoblast cells, cell line Nucleofector Kit V (Lonza, cat# VCA-1003, Gaithersburg, MD) was used.

### DNA constructs

Plasmid constructs expressing the transcription factors were generated with primers 5′- tttCTCGAGatgttaccgacccaagctgg-3′ and 5′-ggggAAGCTTtcattcactcttggtgctgt-3′ for SRF, primers 5′- ttCTCGAGccagactcttcgcagatgtt-3′ and 5′- ccAAGCTTtcactttctgtgcttctcctc-3′ for TFAP2, and primers 5′- ccCTCGAGatgagcgaccaagatcactc-3′ and 5′-ccAAGCTTtcagaagccattgccactga -3′ for SP1. The PCR products were cloned into plasmid pcDNA3.1(-) with *Xho*I and *Hind*III restriction sites. The plasmids for luciferase assay were constructed by cloning PCR products (558 bp (construct I), 228 bp (II), 980 bp (III), and 650 bp (IV)), covering the direct upstream of human *FXN* gene coding sequence or lengthening into the first intron 257 bp of *FXN* gene, into pGL4.10 (Promega, Madison, WI). Site-directed mutagenesis was carried out by using primers 5′- GCAGGCTCTGGATTCGCGTTAAATGCACG -3′ and 5′- CGTGCATTTAACGCGAATCCAGAGCCTGC -3′, or primers 5′- CTGCTCCCCCACAGAAGAGTAAATGCAACCAGTGGCCACCAGG -3′ and 5′- CCTGGTGGCCACTGGTTGCATTTACTCTTCTGTGGGGGAGCAG-3′, or primers 5′- CGCAGCACCAGCGCTGGAAAACAAAGCGGAGCGGGCGGCAGA-3′ and 5′- TCTGCCGCCCGCTCCGCTTTGTTTTCCAGCGCTGGTGCTGCG-3′, or primers 5′- GAGGCGCCGCGCTATTCGGGGTCGCTC-3′ and 5′- GAGCGACCCCGAATAGCGCGGCGCCTC -3′, which contained the mutated predicted binding sites of SRF, TFAP2, SP1, or EGR3, respectively, following the supplier's instructions (Stratagene, La Jolla, CA). Plasmid DNA was isolated with Qiagen miniprep or midiprep kits (Valencia, CA) as needed.

### Chromatin immunoprecipitation (ChIP)

ChIP was conducted with Magna ChIP™ G Kit (Millipore, cat#MAGNA0002, Billerica, MA) following the manufacturer's instruction. Briefly, cells were treated with formaldehyde (1%) for 10 min to crosslink the chromatin with the potential transcription factors. Harvested cells were sonicated to shear chromosomal DNA to an average length between 200 to 600 base pairs. Isolated chromatin was incubated with the proper primary antibody against human transcription factor SRF, TFAP2, SP1, or EGR3 (Santa Cruz Biotech. Inc., Santa Cruz, CA). After a series of wash steps, the chromatin was eluted for further real-time PCR analysis.

### Electrophoretic mobility shift assays (EMSAs)

DNA gel-shifts were carried out with a double-stranded oligonucleotide 201 (oligo 201) 5′- CGTGCATTTAACAAAAATGGAGAGCCTGCTTT-3′ for transcription factor SRF, oligo 202 5′- CAGAAGAGTGCCTGCGGCCAGTGGCCACCA-3′ for transcription factor TFAP2, oligo 203 5′- CCAGCGCTGGAGGGCGGAGCGGGCGGCAGA-3′ for transcription factor SP1 in 20 µl of 25 mM Hepes (pH 7.5), 40 mM NaCl, 1 mM EDTA, 4 mM DTT, and 10% glycerol for 1 hour at 4°C, after labeling the double stranded oligonucleotide with [γ-P^32^]-ATP (Perkin Elmer, cat#NEG035C, Waltham, MA). Nuclear extracts were prepared from HEK293 cells using NE-PER Nuclear Extraction Reagents (Pierce, Rockford, IL, USA) according to the supplier's instructions. For assessment of binding specificity, poly(dI-dC) (Sigma, cat#P4925) was added to each reaction, from a stock concentration of 0.5 mU/µl. Antibody supershift reactions were performed following manufacturers instructions (Active Motif, Carlsbad, CA). Antibodies of SRF and TFAP2 and Jurkat nuclear extract for supershift were purchase from Active Motif. Following 5% native polyacrylamide gel electrophoresis (29:1, acrylamide:bisacrylamide) with Tris-Borate-EDTA (1X TBE) casting and running buffer, gels were dried and exposed to a phosphor screen, which was visualized using a Typhoon Imager (GE, Piscataway, NJ).

### Real-Time PCR

The comparative C_t_ method with SYBR Green was conducted with the ABI 7000 Real-Time PCR System (Applied Biosystems, Foster City, CA). For human *FXN* gene detection, the following primers were used: 5′-CCTTGCAGACAAGCCATACA-3′ and 5′-CCACTGGATGGAGAAGATAG-3′ [21]; for SRF, primer 249 5′-AGAAGGCCTATGAGCTGTCC-3′ and primer 250 5′-TTGCCGGTCTCACTGGTGAT-3′; for TFAP2, primer 274 5′-TCCAACAGCAATGCCGTCTC-3′ and primer 275 5′- GCCACCGTGACCTTGTACTT-3′; for EGR3, primer 265 5′-GCCAGGACAACATCATTAGC-3′ and primer 266 5′-AGGTCGCCGCAGTTGGAGTA-3′. Endogenous GAPDH was used as an internal control with primers 5′-TGCACCACCAACTGCTTAGC-3′ and 5′-GGCATGGACTGTGGTCATGAG-3′ for normalization. Different primer combinations for transcription factor binding sites in ChIP assays were primer 204 5′- TTACACAAGGCATCCGTCTC-3′ and primer 205 5′- GGAGCAGCTAGAGGTTAGAC-3′; primer 206 5′- GGTCTAACCTCTAGCTGCTC-3′ and primer 195 5′- TTTTTTAAGCTTCTGCTCCGGGTCTGCCGCCC-3′; primer 189 5′- ATTGGTACCACCAGGCTGCAGTCTCCCTT-3′ and primer 195; primer 364 5′-TAAGTATCCGCGCCGGGAAC-3′ and primer 365 5′-AATGCAACCGGGAGAACCAG-3′ with template DNA from isolated chromatin (see ChIP experiment) for quantitative real-time PCR. A part of sequence of Human *FXN* intron 4 was amplified as a ChIP negative internal control with primers 5′-AGGCCAAGGCCTGTGGATCA-3′ and 5′-AGTGGCGCGATCTTGGCTCA-3′.

### Western Blotting Analysis

Proteins were resolved in 12% NUPAGE gels (Invitrogen, cat# NP0342box) and transferred onto nitrocellulose membranes (Invitrogen, cat# IB3010-01). Primary antibodies used were rabbit anti-TFAP2 and anti-SP1 (Sant Cruz Biotech, Inc, Santa Cruz, CA), mouse anti-SRF and anti-tubulin (Abcam, Cambridge, MA), and mouse anti-frataxin (MitoScience, Eugene, Oregon). The mature frataxin form (∼14 kDa) was quantified for comparison when needed. Western blot band intensities were quantified using program ImageJ. Any change of the intensities was compared with the controls, which value was set as 1.

### Luciferase assay

The indicated plasmids derived from pGL4.10 [luc2] (Promega) were co-transfected with pGL4.75 [hRluc/CMV] into HEK293, K562, or SH SY5Y cells. Luciferase activities were measured with Dual-Luciferase® Reporter Assay System (Promega) by using Veritas microplate luminometer (Turner Biosystems, Sunnyville, CA). For control of transfection efficiency in each well, firefly luciferase activity was normalized to *Rellina* luciferase activity.

### Bioinformatic and statistical analysis

Online software Genomatix (http://www.genomatix.de) was used to search the binding sites of putative transcription factors in the promoter region of the human *FXN* gene. Other statistical analyses in this study were performed using the Student's *t*-test (http://www.danielsoper.com/statcalc/).

## References

[1] Sanchez-Casis G, Cote M, Barbeau A (1976). Pathology of the heart in Friedreich's ataxia: review of the literature and report of one case.. Can J Neurol Sci.

[2] Babcock M, de Silva D, Oaks R, Davis-Kaplan S, Jiralerspong S (1997). Regulation of mitochondrial iron accumulation by Yfh1p, a putative homolog of frataxin.. Science.

[3] Rotig A, de Lonlay P, Chretien D, Foury F, Koenig M (1997). Aconitase and mitochondrial iron-sulphur protein deficiency in Friedreich ataxia.. Nat Genet.

[4] Lesuisse E, Santos R, Matzanke BF, Knight SA, Camadro JM (2003). Iron use for haeme synthesis is under control of the yeast frataxin homologue (Yfh1).. Hum Mol Genet.

[5] Gakh O, Adamec J, Gacy AM, Twesten RD, Owen WG (2002). Physical evidence that yeast frataxin is an iron storage protein.. Biochemistry.

[6] Yoon T, Cowan JA (2003). Iron-sulfur cluster biosynthesis. Characterization of frataxin as an iron donor for assembly of [2Fe-2S] clusters in ISU-type proteins.. J Am Chem Soc.

[7] Ramazzotti A, Vanmansart V, Foury F (2004). Mitochondrial functional interactions between frataxin and Isu1p, the iron-sulfur cluster scaffold protein, in Saccharomyces cerevisiae.. FEBS Lett.

[8] Schulz JB, Boesch S, Burk K, Durr A, Giunti P (2009). Diagnosis and treatment of Friedreich ataxia: a European perspective.. Nat Rev Neurol.

[9] Kakhlon O, Manning H, Breuer W, Melamed-Book N, Lu C (2008). Cell functions impaired by frataxin deficiency are restored by drug-mediated iron relocation.. Blood.

[10] Richardson DR, Mouralian C, Ponka P, Becker E (2001). Development of potential iron chelators for the treatment of Friedreich's ataxia: ligands that mobilize mitochondrial iron.. Biochim Biophys Acta.

[11] Hart PE, Lodi R, Rajagopalan B, Bradley JL, Crilley JG (2005). Antioxidant treatment of patients with Friedreich ataxia: four-year follow-up.. Arch Neurol.

[12] Rustin P (2003). The use of antioxidants in Friedreich's ataxia treatment.. Expert Opin Investig Drugs.

[13] Baralle M, Pastor T, Bussani E, Pagani F (2008). Influence of Friedreich ataxia GAA noncoding repeat expansions on pre-mRNA processing.. Am J Hum Genet.

[14] Al-Mahdawi S, Pinto RM, Ismail O, Varshney D, Lymperi S (2008). The Friedreich ataxia GAA repeat expansion mutation induces comparable epigenetic changes in human and transgenic mouse brain and heart tissues.. Hum Mol Genet.

[15] Greene E, Mahishi L, Entezam A, Kumari D, Usdin K (2007). Repeat-induced epigenetic changes in intron 1 of the frataxin gene and its consequences in Friedreich ataxia.. Nucleic Acids Res.

[16] Rai M, Soragni E, Jenssen K, Burnett R, Herman D (2008). HDAC inhibitors correct frataxin deficiency in a Friedreich ataxia mouse model.. PLoS One.

[17] Soragni E, Herman D, Dent SY, Gottesfeld JM, Wells RD (2008). Long intronic GAA*TTC repeats induce epigenetic changes and reporter gene silencing in a molecular model of Friedreich ataxia.. Nucleic Acids Res.

[18] Grabczyk E, Usdin K (2000). The GAA*TTC triplet repeat expanded in Friedreich's ataxia impedes transcription elongation by T7 RNA polymerase in a length and supercoil dependent manner.. Nucleic Acids Res.

[19] Grabczyk E, Usdin K (2000). Alleviating transcript insufficiency caused by Friedreich's ataxia triplet repeats.. Nucleic Acids Res.

[20] Krasilnikova MM, Kireeva ML, Petrovic V, Knijnikova N, Kashlev M (2007). Effects of Friedreich's ataxia (GAA)n*(TTC)n repeats on RNA synthesis and stability.. Nucleic Acids Res.

[21] Li K, Besse EK, Ha D, Kovtunovych G, Rouault TA (2008). Iron-dependent regulation of frataxin expression: implications for treatment of Friedreich ataxia.. Hum Mol Genet.

[22] Greene E, Entezam A, Kumari D, Usdin K (2005). Ancient repeated DNA elements and the regulation of the human frataxin promoter.. Genomics.

[23] Knoll B, Nordheim A (2009). Functional versatility of transcription factors in the nervous system: the SRF paradigm.. Trends Neurosci.

[24] Eckert D, Buhl S, Weber S, Jager R, Schorle H (2005). The AP-2 family of transcription factors.. Genome Biol.

[25] Mao XR, Moerman-Herzog AM, Chen Y, Barger SW (2009). Unique aspects of transcriptional regulation in neurons–nuances in NFkappaB and Sp1-related factors.. J Neuroinflammation.

[26] Sysa P, Potter JJ, Liu X, Mezey E (2009). Transforming growth factor-beta1 up-regulation of human alpha(1)(I) collagen is mediated by Sp1 and Smad2 transacting factors.. DNA Cell Biol.

[27] Reid LH, Gregg RG, Smithies O, Koller BH (1990). Regulatory elements in the introns of the human HPRT gene are necessary for its expression in embryonic stem cells.. Proc Natl Acad Sci U S A.

[28] Smith AN, Barth ML, McDowell TL, Moulin DS, Nuthall HN (1996). A regulatory element in intron 1 of the cystic fibrosis transmembrane conductance regulator gene.. J Biol Chem.

[29] Antoine M, Kiefer P (1998). Functional characterization of transcriptional regulatory elements in the upstream region and intron 1 of the human S6 ribosomal protein gene.. Biochem J.

[30] Brudno M, Gelfand MS, Spengler S, Zorn M, Dubchak I (2001). Computational analysis of candidate intron regulatory elements for tissue-specific alternative pre-mRNA splicing.. Nucleic Acids Res.

[31] Campuzano V, Montermini L, Lutz Y, Cova L, Hindelang C (1997). Frataxin is reduced in Friedreich ataxia patients and is associated with mitochondrial membranes.. Hum Mol Genet.

[32] Campuzano V, Montermini L, Molto MD, Pianese L, Cossee M (1996). Friedreich's ataxia: autosomal recessive disease caused by an intronic GAA triplet repeat expansion.. Science.

[33] Montermini L, Richter A, Morgan K, Justice CM, Julien D (1997). Phenotypic variability in Friedreich ataxia: role of the associated GAA triplet repeat expansion.. Ann Neurol.

[34] Lee JG, Dahi S, Mahimkar R, Tulloch NL, Alfonso-Jaume MA (2005). Intronic regulation of matrix metalloproteinase-2 revealed by in vivo transcriptional analysis in ischemia.. Proc Natl Acad Sci U S A.

[35] van Helden J, Rios AF, Collado-Vides J (2000). Discovering regulatory elements in non-coding sequences by analysis of spaced dyads.. Nucleic Acids Res.

[36] Tourtellotte WG, Milbrandt J (1998). Sensory ataxia and muscle spindle agenesis in mice lacking the transcription factor Egr3.. Nat Genet.

[37] Carter JH, Lefebvre JM, Wiest DL, Tourtellotte WG (2007). Redundant role for early growth response transcriptional regulators in thymocyte differentiation and survival.. J Immunol.

